# Impact of Antenatal SARS-CoV-2 Exposure on SARS-CoV-2 Neutralization Potency

**DOI:** 10.3390/vaccines12020164

**Published:** 2024-02-05

**Authors:** Chia-Jung Chiang, Wei-Lun Hsu, Mei-Tsz Su, Wen-Chien Ko, Keng-Fu Hsu, Pei-Yin Tsai

**Affiliations:** 1 Department of Obstetrics and Gynecology, National Cheng Kung University Hospital, College of Medicine, National Cheng Kung University, Tainan 704302, Taiwan; 2 Department of Internal Medicine, National Cheng Kung University Hospital, College of Medicine, National Cheng Kung University, Tainan 704302, Taiwan; 3 Center for Infection Control, National Cheng Kung University Hospital, College of Medicine, National Cheng Kung University, Tainan 704302, Taiwan

**Keywords:** vaccines, COVID-19, SARS-CoV-2, pregnant women, infants, immune response, neutralizing antibody

## Abstract

A pregnancy booster dose significantly reduces the risk and severity of COVID-19, and it is widely recommended. A prospective cohort study was conducted to compare the transplacental passage of maternal antibodies from vaccination or infection during three trimesters against both the vaccine-targeted Wuhan strain and the Omicron strain of SARS-CoV-2. Maternal–infant dyads from vaccinated mothers were collected between 6 June 2022 and 20 September 2022. We analyzed 38 maternal–infant dyads from mothers who had been infected with COVID-19 and 37 from mothers without any previous infection. Pregnant women who received their last COVID-19 vaccine dose in the third trimester exhibited the highest anti-spike protein antibody levels and neutralizing potency against both the Wuhan strain and Omicron BA.2 variant in their maternal and cord plasma. Both second- and third-trimester vaccination could lead to a higher level of neutralization against the Wuhan and Omicron strains. COVID-19 infection had a negative effect on the transplacental transfer ratio of SARS-CoV-2 antibodies. A booster dose during the second or third trimester is encouraged for the maximum transplacental transfer of humoral protection against COVID-19 for infants.

## 1. Introduction

The global outbreak of severe acute respiratory syndrome coronavirus 2 (SARS-CoV-2) since December 2019 has been a major public health concern. Taiwan, an island in East Asia, had a low incidence of coronavirus disease 2019 (COVID-19) until 2020 [1,2]. In April 2022, an island-wide surge due to the Omicron variant (BA.2.3.7) was noted [3].

The safety and efficacy of COVID-19 vaccination during pregnancy has been supported by contemporary evidence [4,5,6]. Since no COVID-19 vaccine was available for neonates, pregnant individuals were encouraged to receive the COVID-19 vaccines due to the increased risk of adverse maternal outcomes in antepartum COVID-19 [7,8,9], and the evidence that it could provide neonatal protection for up to 6 months after birth [10]. The transplacental transfer of SARS-CoV-2 antibodies, specifically against the receptor-binding domain (RBD) of spike protein (anti-S) and nucleocapsid antigen (anti-N), has been observed in previous studies of infected pregnant women or those vaccinated against COVID-19 [11,12,13,14].

Various maternal vaccination strategies have been proposed to maximize neonatal protection [15,16]. Some studies suggest starting the primary series in the early third trimester (27–31 weeks) [17], while others suggest a final dose of the primary series after 31 weeks [10]. Additionally, three doses during pregnancy, including a third-trimester booster, are recommended [18]. A pregnancy booster dose significantly reduces the risk and severity of COVID-19, and it is widely recommended [18,19,20,21,22]. The World Health Organization has proposed a mid-second-trimester booster dose [21], but comparisons of booster effects across different trimesters regarding infection status and neutralization potency are still insufficient.

Given that a booster dose during pregnancy is being widely accepted, we aim to compare the transplacental passage and neutralization effect of maternal antibodies from vaccination or infection during three trimesters against both the vaccine-targeted Wuhan strain and the Omicron strain of SARS-CoV-2. We focused on the change in the transplacental transfer of antibodies and neutralization in vaccinated pregnant women with or without a primary Omicron infection.

## 2. Materials and Methods

A prospective cohort study was conducted at National Cheng Kung University Hospital (NCKUH), a tertiary center in Taiwan, between 28 June 2022 and 20 September 2022. Eligible participants included pregnant women who met all the following criteria: (1) women who delivered at term, (2) women who received at least one dose of COVID-19 vaccine, (3) women with or without laboratory-confirmed primary COVID-19 during pregnancy, (4) women with samples of cord blood and their blood available within three days after delivery, and (5) women who provided informed consent. Exclusion criteria included (1) those without any COVID-19 vaccine, (2) those with more than one episode of COVID-19, or (3) those with history of COVID-19 before pregnancy.

The participants were categorized by the trimester of their last vaccination, i.e., the third (T3, ≥27 weeks), second (T2, 13–27 weeks), and first (T1, <13 weeks) trimesters. The diagnosis of COVID-19 was confirmed by real-time polymerase chain reaction (RT-PCR) or rapid antigen testing of respiratory tract specimens. The study was approved by the Institutional Review Board of NCKUH (IRB: B-ER-111-164) and all participants provided informed consent.

Clinical and laboratory data were collected from electronic medical records in a pre-determined form. Cord blood was obtained from the umbilical cord immediately after delivery. Blood samples were centrifuged at 1000× *g* for 10 min at room temperature, and plasma samples were aliquoted into dedicated pre-coded tubes and stored at –80 °C until analysis.

Antibody levels of SARS-CoV-2 in the maternal and cord blood were measured by the Elecsys^®^ Anti-SARS-CoV-2 S assay (Roche Diagnostics, Rotkreuz, Switzerland). Antibodies against nucleocapsid (anti-N) and receptor-binding domain (RBD) of spike antigen (anti-S RBD) of SARS-CoV-2 were detected. The cut-off for positivity was ≥1.0 U/mL for anti-N antibody and ≥0.8 U/mL for anti-S RBD antibody. The samples with an anti-S RBD concentration of >250 U/mL were diluted by 10- or 100-fold and measured again for the anti-S RBD. The cPass^TM^ Surrogate Virus Neutralization Detection kit (GenScript Biotech Corp., Piscataway, NJ, USA) was used to measure neutralizing antibody (nAb) titers against two SARS-CoV-2 variants. Ninety-six-well plates coated with recombinant hACE2 and recombinant S-RBD from the Wuhan strain and Omicron BA.2 variant were used in the experiments, according to the manufacturer’s instructions. An inhibition percentage of ≥30% was considered to be seropositive for SARS-CoV-2 nAbs [23].

Categorical variables between two cohorts were compared by the chi-square test with a two-sided *p* value, or the Fisher’s exact test if a cell’s expected count was less than 5. Continuous parameters were compared by the independent sample *t* test, or non-parametric Mann–Whitney U test if lack of normality was noted by skewness and kurtosis above 2, respectively [24]. Data were summarized as means and standard deviation (SDs) or medians and quartile deviation (QDs). A *p* value of <0.05 was considered significant.

The participants were categorized based on the trimester of their last COVID-19 vaccination, i.e., the third (T3, ≥27 weeks), second (T2, 13–27 weeks), and first trimesters (T1, <13 weeks) and before pregnancy. Effects of maternal COVID-19 infection were also analyzed by infection status, grouping the maternal–fetal dyads into COVID-19 negative, with COVID-19 noted within 7 days, and with COVID-19 noted for more than 7 days.

Normality analysis for univariate outcomes in each group was conducted, with a criterion of skewness and kurtosis below 2 to meet the normality assumption [24]. Comparisons of anti-S RBD antibody and neutralizing antibody (nAb) concentrations across groups, categorized based on trimester of last vaccination and infection status, were analyzed using one-way ANOVA tests and post-hoc Bonferroni tests if the normality assumption was met. In cases where normality was not assumed, Kruskal–Wallis tests followed by post-hoc Mann–Whitney U tests were employed.

Data distribution was visualized as violin plots and box plots to show humoral responses in pregnant women receiving the latest injection of a COVID-19 vaccine during different trimesters, and to show the effect of COVID-19 infection at various timings. Dots and interconnection between the samples from maternal–fetal dyads are shown to express the correlations.

A multivariable linear regression analysis was employed to examine the association among various factors, including prior history of COVID-19 during pregnancy and COVID-19 vaccines administered in each trimester; their impact on plasma levels and transplacental transfer ratio of anti-S RBD; and also nAb against the Wuhan strain and Omicron BA.2 variant. The selection of the eligible variables was guided by the previous literature [23]. The absence of collinearity among the variables was confirmed by the variance inflation factor, which remained below the threshold of 2.0. The equal variance assumption for the variates was confirmed using the Durbin–Watson test, with results ranging from 1.989 to 2.342.

The normality of our six outcomes from sera including maternal anti-S RBD antibody, cord anti-S RBD antibody, maternal Wuhan nAb, cord Wuhan nAb, maternal Omicron nAb, and cord Omicron nAb were explored by skewness and kurtosis [25]. Skewness fell between −1.771 and 2.847, and kurtosis fell between −1.160 and 9.657, meeting the normality assumption for the six outcomes in multivariable analysis [26,27,28].

Time-correlation of anti-S antibody in maternal or cord blood was plotted as scatter plots, with log_10_ transformation done for anti-S antibody levels. The time interval was defined as the period between the most recent administration of a COVID-19 vaccine or occurrence of a COVID-19 infection and the time of blood sampling. To investigate the temporal dynamics of antibody concentrations concerning the interval from antigen exposure to delivery, quadratic curves were plotted using a polynomial regression approach to address the non-linear temporal dynamics seen in previous clinical observations [29,30]. Patients infected within 7 days were excluded from the regression as the prior univariable analysis revealed no significant antibody differences.

Time-correlations of neutralization potency against Wuhan or Omicron strains in maternal or cord blood were plotted as scatter plots. The time interval was defined as the period between the most recent administration of a COVID-19 vaccine and the time of blood sampling. To investigate the temporal dynamics of neutralization potency after vaccination, linear regression was utilized for visualization, chosen from previous clinical observations [29].

The transplacental transfer ratio was calculated as the antibody concentration in cord blood divided by that in maternal blood, indicative of the efficacy of transplacental transfer of antibodies. Normality analysis for anti-S transfer ratio for all maternal-fetal dyads yielded 0.675 for skewness and 0.463 for kurtosis. A multivariable linear regression analysis was then employed for all maternal–infant dyads to examine the association among prior history of COVID-19 during pregnancy, COVID-19 vaccines administered in each trimester, and their impact on transplacental transfer ratios of anti-S antibody.

Time variable was presented as the time interval from delivery to the last antigen exposure, either the last vaccination or COVID-19 infection, whichever occurred last. Transfer ratios of antibodies were visualized as scattered plots and plotted as quadratic curves by polynomial regression approach against the time interval from last antigen exposure, to address the non-linear temporal dynamics seen in previous clinical observations [14].

Post-hoc power analyses were conducted for the multivariable linear regressions. The six regression models, representing maternal anti-S antibody, cord anti-S antibody, maternal Wuhan neutralization, cord Wuhan neutralization, maternal Omicron neutralization, and cord Omicron neutralization, respectively, demonstrate a power range ranging from 66.0% to 99.9%.

Statistical analyses were conducted by the SPSS version 28.0 and Prism version 9 (GraphPad, La Jolla, CA, USA).

## 3. Results

### 3.1. Maternal and Infant Characteristics

**Figure 1:**
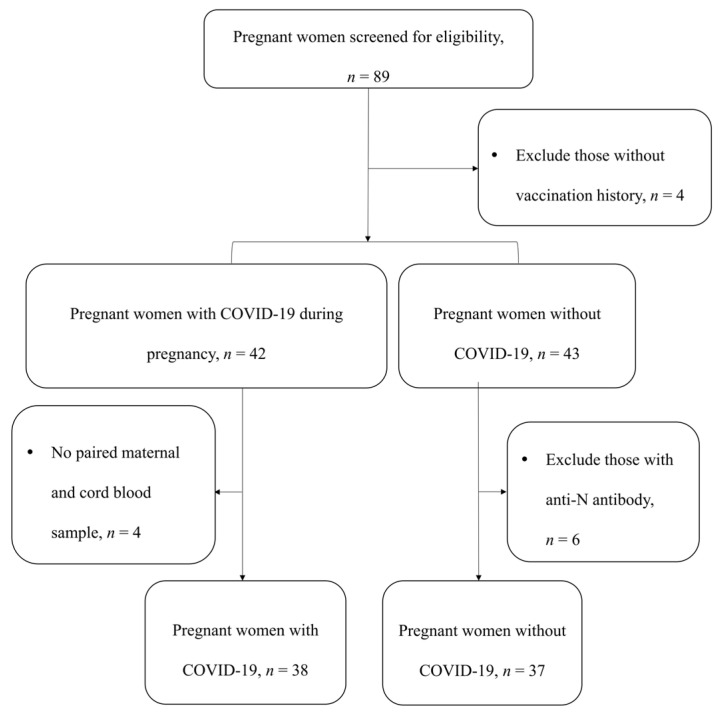
Flow-Chart of Study Population. Abbreviations: COVID-19, coronavirus disease (COVID-19); Anti-N, anti-nucleocapsid antibody.

Between 6 June 2022 and 20 September 2022, there were 89 women enrolled in this study. Four unvaccinated women were excluded. Of these, 42 women were either previously diagnosed with COVID-19 during pregnancy or had a positive RT-PCR result of SARS-CoV-2 in nasopharyngeal samples upon admission. Four women had no paired samples of maternal and cord blood and were excluded as a result. There were 43 women without a prior history of COVID-19. Six women were excluded due to the detection of the anti-N antibody, indicating previous unrecognized asymptomatic COVID-19. Ultimately, 75 maternal–cord pairs were included for analysis, consisting of 38 pairs from those with prior or current COVID-19 (the case group) and 37 pairs from those without COVID-19 (the control group) (Figure 1).

Demographic, obstetric, and clinical characteristics of the participants are summarized in Table 1. Both groups had similar profiles in terms of their age, body mass index (BMI), gravidity, and gestational age at birth. The Cesarean section rate was higher in the control group (49% vs. 21%, *p* = 0.012), as they were often admitted to the tertiary center because of high-risk pregnancy.

Our study focused on a highly vaccinated pregnant population. Seventy-two participants (96%) had received one or more vaccinations during pregnancy. Thirty-nine participants (52%) had completed a primary series before pregnancy. Forty-four participants (59%) had received a booster dose during various trimesters. All of the participants received first-generation vaccines designed for the Wuhan strain.

All participants included in the analysis were vaccinated, and 44 (58.7%) of the women had completed a primary series with one booster dose. Eighty-five vaccine doses were administered during pregnancy in our cohort, including 24 (27.0%) doses administered during the third trimester, 36 (42.3%) doses during the second trimester, and 25 (29.4%) doses during the first trimester. The vaccines administered during pregnancy included 72 doses of (84.7%) mRNA vaccines (NT162b2, Pfizer/BioNTech or mRNA-1273, Moderna), 11 doses (12.9%) of a viral vector vaccine (ChAdOx1 nCoV-19, Oxford-AstraZeneca), and 2 (2.4%) doses of a protein subunit vaccine (MVC-COV1901, Medigen). The differences in the intervals from the last vaccination to delivery were not significant in the case (mean intervals: 145.7 days) and control (143 days) groups (*p* = 0.902) (Table 1).

Participants from the case and control groups showed similar characteristics in terms of their vaccination history and type. Most of the women (82.7%) received mRNA vaccines during pregnancy. Notably, 11 doses of viral vector vaccines were administered during pregnancy, all of which were second doses to complete a primary series. Among them, seven doses were given during the first trimester before the pregnant women became aware of their pregnancy, and four doses were given during the second trimester. No adverse event was reported among all of the women and their offspring.

### 3.2. Anti-S Antibody Response and Neutralization Potency in Three Trimesters of Pregnant Vaccines

**Figure 2:**
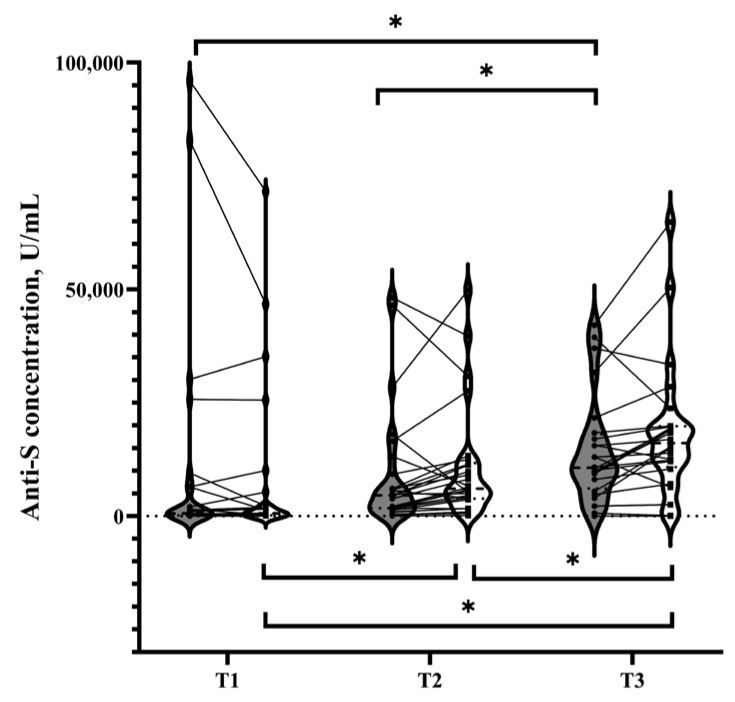
Violin plots and dot plots show humoral responses in pregnant women receiving the latest injection of a COVID-19 vaccine during different trimesters: anti-S antibody concentrations of maternal (shown in gray; ●) and cord (shown in white; ■) blood in all pregnant women. Statistical significance was determined by the Mann–Whitney U test. An asterisk (*) indicates a *p* value of <0.017. Abbreviations: T1, before pregnancy and during the first trimester; T2, during the second trimester; T3, during the third trimester; Anti-S, anti-spike protein antibody.

The participants were categorized based on the trimester of their last COVID-19 vaccination, i.e., the third (T3, ≥27 weeks), second (T2, 13–27 weeks), and first trimesters (T1, <13 weeks) and before pregnancy. Among all participants, the highest concentration of anti-S antibodies was observed in those receiving the latest vaccine during the third trimester (T3 group) in maternal and cord blood (median 10,627.50 U/mL, IQR 6102.75–18,021.50 U/mL in maternal plasma; median 16,082.50, IQR 10,805.50–19,823.75 U/mL in cord plasma) when compared to T1 (median 578.50 U/mL, IQR 160.25–6425.00 U/mL for maternal plasma, *p* value < 0.001; median 576.10 U/mL, IQR 184.00–3757.00 U/mL for cord plasma, *p* value < 0.001) and T2 (median 4565.00 U/mL, IQR 1749.00–10,213.25 U/mL for maternal plasma, *p* value = 0.013; median 6017.00 U/mL, IQR 3838.75–11,717.25 U/mL for cord plasma, *p* value = 0.006) groups (Figure 2). A higher concentration of Anti-S antibodies was also observed in participants receiving the latest vaccine in the T2 group than in the T1 group for the cord plasma (*p* = 0.002).

**Figure A1:**
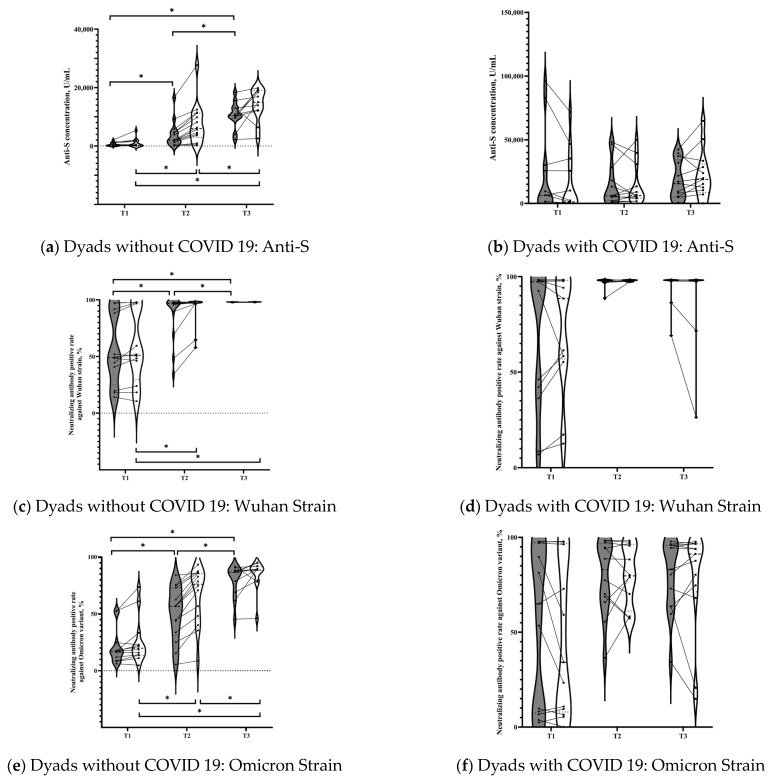
Violin plots and dot plots show humoral responses in pregnant women receiving the latest injection of a COVID-19 vaccine during different trimesters: anti-S antibody concentrations of maternal (shown in gray; ●) and cord (shown in white; ■) in pregnant women without COVID-19 (**a**), or those with COVID-19 (**b**); neutralizing antibody concentrations of maternal and cord blood against the Wuhan strain in pregnant without COVID-19 (**c**), or those with COVID-19 (**d**); neutralizing antibody concentrations against the Omicron variant in pregnant women without COVID-19 (**e**), or those with COVID-19 (**f**). Statistical significance was determined by the Mann-Whitney U test or the Bonferroni test. An asterisk (*) indicates a *p* value of <0.017. Abbreviations: T1, before pregnancy and during the first trimester; T2, during the second trimester; T3, during the third trimester; COVID-19, coronavirus disease (COVID-19); Anti-S, anti-spike protein antibody.

For the control group, the anti-S antibody titers of the maternal and cord blood were significantly higher in the T3 group (median 10,442.00 U/mL, IQR 9535.00–13,033.00 U/mL for maternal plasma; median 14,999.00 U/mL, IQR 12,085.00–19,042.00 U/mL for cord plasma) than in the T1 (median 264.15 U/mL, IQR 56.60–1075.98 U/mL for maternal plasma, *p* value < 0.001; median 269.80 U/mL, IQR 75.54–1819.75 U/mL for cord plasma, *p* value < 0.001) and T2 (median 2035.50 U/mL, IQR 1070.93–4912.75 U/mL for maternal plasma, *p* value < 0.001; median 5937.50 U/mL, IQR 2932.33–11,280.75 U/mL for cord plasma, *p* value = 0.006) groups, as shown in Figure A1a. However, the significance of the vaccination trimester regarding the anti-S antibody titers of maternal or cord blood was diminished in the case group (Figure A1b), which suggests a prominent impact of recent COVID-19 on the anti-S antibody titers (Tables S1 and S2).

**Figure 3:**
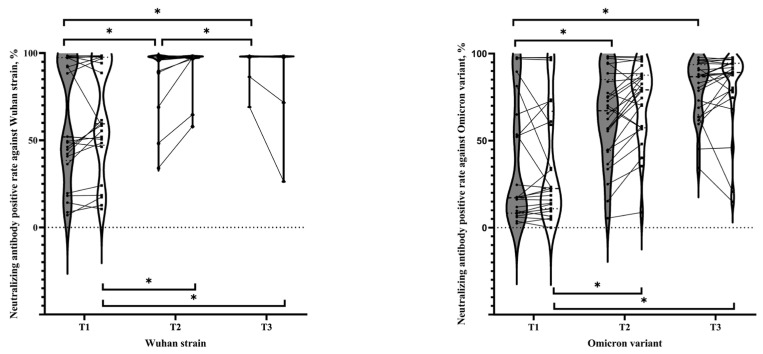
Violin plots and dot plots show humoral responses in pregnant women receiving the latest injection of a COVID-19 vaccine during different trimesters: neutralizing antibody concentrations of maternal (shown in gray; ●) and cord (shown in white; ■) blood against the Wuhan strain and Omicron variant in all pregnant women. Statistical significance was determined by the Mann–Whitney U test or the Bonferroni test. An asterisk (*) indicates a *p* value of <0.017. Abbreviations: T1, before pregnancy and during the first trimester; T2, during the second trimester; T3, during the third trimester.

The neutralization response rates grouped by the last vaccination at three trimesters were similar to those of the anti-S antibodies. For all maternal–fetal dyads, maximum neutralizing responses against the wild-type Wuhan strain and Omicron BA.2 variant were present in the T3 group (median 98.15%, IQR 98.05–98.22% and median 98.15%, IQR 98.01–98.22% in the maternal and cord sera for the Wuhan strain, respectively; median 86.71%, IQR 65.02–93.69% and median 89.12%, IQR 78.27–94.45% in the maternal and cord sera for the Omicron strain, respectively) (Figure 3). The neutralizing responses of COVID-19 vaccines against the Omicron BA.2 variant were more pronounced in the control participants (T3 group of cord sera with median 98.15%, IQR 98.04–98.22% for the Wuhan strain; median 88.94, IQR 79.36–91.98% for the Omicron strain) (Figure A1c,e).

### 3.3. Multivariable Linear Regression for Anti-S Response and Neutralization Potency

A multivariate linear regression analysis was employed to examine the association between a history of COVID infection during pregnancy or vaccinations administered in each trimester, and their respective impact on the anti-S levels and neutralization potency for the Wuhan and Omicron strains. 

Among the potential factors affecting the anti-S antibody levels in pregnant women, COVID-19 during pregnancy was the only significant predictor, estimated to increase the maternal anti-S antibody level by 1096.887U/mL (*p* = 0.006). In contrast, COVID-19 vaccination during the third trimester (estimated to increase cord anti-S antibody by 9838.372U/mL, *p* value = 0.013) and COVID-19 during pregnancy (estimated to increase cord anti-S antibody by 7797.896U/mL, *p* value = 0.026) were significantly associated with the anti-S antibody levels in cord blood. Therefore, only a third-trimester COVID-19 vaccine was associated with a higher anti-S antibody level in cord blood (Table 2).

COVID-19 vaccines given during both the second and the third trimester were associated with an increase in neutralization response rates in maternal and cord blood to the Wuhan strain and Omicron BA.2 variant (estimated effect on response rate of maternal and cord blood’s neutralizing potency around 18.330–37.060%, all *p* values < 0.05). In addition, vaccination during the first trimester could boost the neutralization potency, with an estimated 17.383% increase in the response rate against the Omicron BA.2 variant in cord blood (*p* value = 0.021) (Table 2).

Maternal Omicron infection significantly boosts the Omicron BA.2-specific neutralization antibodies in maternal blood (estimated effect on response rate 16.150%, *p* value = 0.015), as well as boosting maternal and cord blood anti-S antibody levels (Table 2). However, maternal Omicron infection was not associated with increased cord neutralization for the Omicron BA.2 variant in our study.

Maternal COVID-19 infection with the Omicron strain during pregnancy was not associated with maternal or cord blood neutralization potency regarding the Wuhan strain. Maternal Omicron infection selectively enhances neutralization titers for the Omicron variant, not the Wuhan strain, which is suggestive of an immune escape from the Wuhan strain during the Omicron wave in our cohort [3].

### 3.4. The Anti-S Antibody Levels and Time Intervals

**Figure 4:**
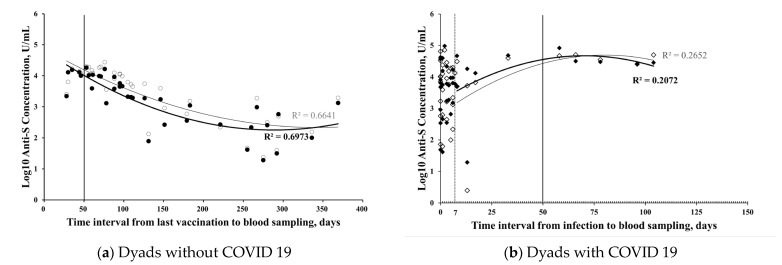
Scatter plots present (**a**) the correlation of transformed log_10_ concentrations of anti-S antibody in maternal (●, R^2^ = 0.6973) or cord blood (○, R^2^ = 0.6641), and the time interval between the latest injection of a COVID-19 vaccine and blood sampling from 37 mothers without SARS-CoV-2 infection; (**b**) the correlation of transformed log_10_ concentrations of anti-S antibody in maternal (◆, R^2^ = 0.2072) or cord blood (◇, R^2^ = 0.2652), and the time interval between the latest injection of a COVID-19 vaccine and blood sampling from 38 mothers with COVID-19.

**Figure A2:**
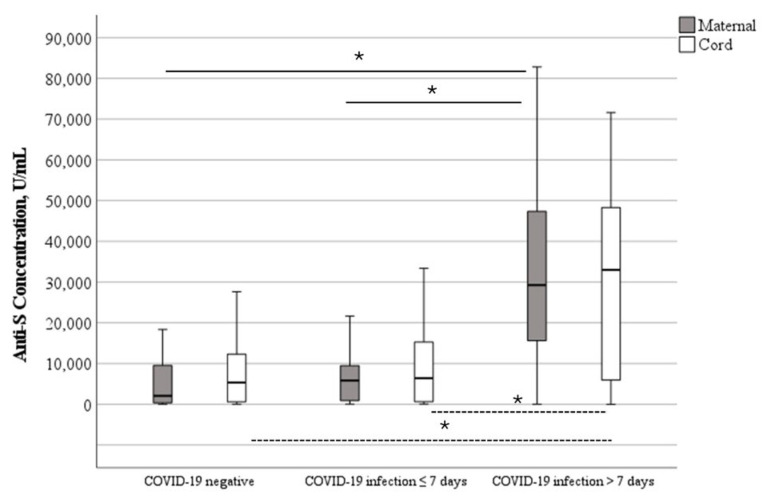
Box plots show the concentrations (U/mL) of anti-spike protein antibody in maternal (shown in gray) and cord blood (shown in white) from maternal-infant dyads without COVID-19, with COVID-19 noted within 7 days, and with COVID-19 noted for more than 7 days. Note: Statistical significance was determined by the Bonferroni test or the Mann-Whitney U test. An asterisk (*) indicates a *p* value of <0.017. Abbreviation: Anti-S, anti-spike protein antibody.

Quadratic curves were plotted using a polynomial regression approach. Those who tested positive for COVID-19 within 7 days before delivery were excluded, as this group showed no significant differences in their anti-S antibody concentrations of maternal and cord blood as compared to the non-infected mothers (Figure A2 and Table S3). The levels of the anti-S antibody concentrations in the maternal blood increased and peaked around 50 days after the last vaccination (Figure 4a) or COVID-19 exposure (Figure 4b). Delayed peaks were observed in the cord blood, as compared with its maternal counterpart. The anti-S antibodies were measurable in maternal and cord blood up to one year after the last vaccination (Figure 4a).

### 3.5. Neutralization Potency for Non-Infected Individuals and Time Relationship

**Figure 5:**
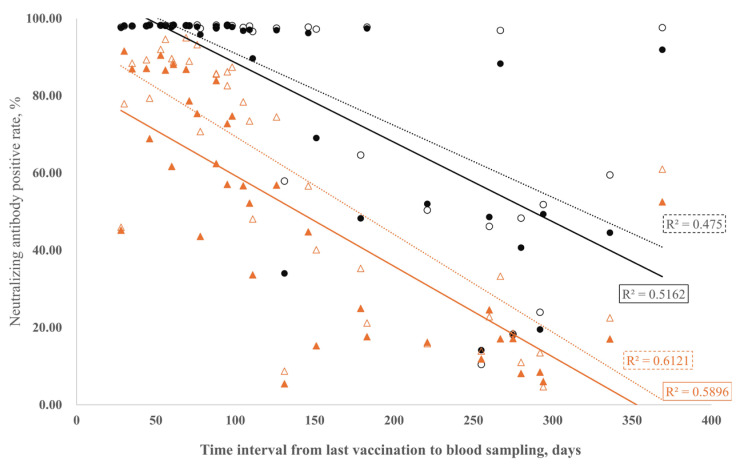
Scatter plots present the correlation of neutralizing antibody positive rate against Wuhan strain in maternal (●, R^2^ = 0.5162) or cord blood (○, R^2^ = 0.475), and the time interval between the latest injection of a COVID-19 vaccine and blood sampling; and the correlation of neutralizing antibody positive rate against Omicron variant in maternal (▲, R^2^ = 0.6121) or cord blood (△, R^2^ = 0.5896), and the time interval between the latest injection of a COVID-19 vaccine and blood sampling from 37 mothers without SARS-CoV-2 infection.

The neutralization potency for non-infected individuals decreased with the time after vaccination. Linear regression was utilized for visualization, as theoretically and clinically assumed by the previous literature [29]. For the first-generation vaccines that were administered, individuals showed a higher neutralization potency for the Wuhan strain than for the Omicron strain (Figure 5). A delayed declined for cord sera when compared to their maternal counterparts was found for both Wuhan and Omicron neutralization, similar to the temporal dynamics of the anti-S antibodies (Figure 4a).

### 3.6. Transplacental Transfer Ratio of Antibodies and Time Relationship

**Figure 6:**
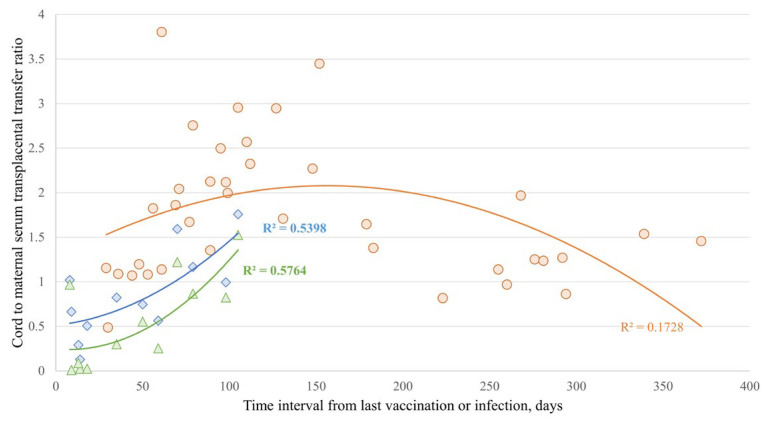
Scatter plots present the correlation of time intervals between the latest injection of a COVID-19 vaccine or the timing of laboratory diagnosis of COVID-19 and the date of delivery, and transplacental ratios of anti-spike protein (S) antibody in 37 pregnant women without SARS-CoV-2 infection (●, R^2^ = 0.1728), or anti-S (◆, R^2^ = 0.5398) and anti-nucleocapsid antibody (▲, R^2^ = 0.5764) in 12 pregnant women diagnosed as having COVID-19 for >7 days.

The transplacental transfer ratios of anti-S antibodies in the case and control groups and anti-N antibodies in the case group showed a positive time correlation for up to 100 days after the antigen exposure, including vaccination or COVID-19 infection. Quadratic curves for each antibody were plotted by the polynomial regression approach for transfer ratios (Figure 6). The transfer ratios of anti-S antibodies from the control group reached 1.0–1.5 around 50 days after antigen exposure by last vaccination, peaked to 2.0–4.0 at 100 days, and then plateaued between 1.0 and 2.0 after 150 days. In contrast, the transfer ratios of the anti-S and anti-N antibodies from the case group were generally below 1.0 within 100 days of antigen exposure by COVID-19 infection (Figure 6).

To pool the effects of antigen exposure from both vaccination and COVID-19 infection, and to examine the association between the history of COVID infection during pregnancy or vaccinations administered in each trimester, and their respective impact on the transplacental ratio of anti-S antibodies, a multivariable linear regression analysis was employed for all maternal–infant dyads. Antepartum COVID-19 exposure was associated with a 0.605 decrease in the transfer ratio (*p* ≤ 0.001). A second-trimester vaccine was associated with a 0.583 increase in the transfer ratio (*p* = 0.001) by a multivariable linear regression model (Table A1).

## 4. Discussion

Our study revealed that neonates born to mothers who received a booster dose of the COVID-19 vaccine during the third trimester, especially those without prior COVID-19 infection, had the highest levels of cord serum anti-S concentration (Figure 2) and the most effective protection against both the Wuhan and Omicron strains by group comparisons (Figure 3). Similarly, higher levels of neonatal anti-S antibodies were found among those who received maternal vaccination after 31 weeks of pregnancy in the previous literature, although without comparison across three trimesters [10]. Our results are consistent with previous experience with the combined tetanus-diphtheria-pertussis (TDP) vaccine. Immunization with the tetanus-diphtheria-pertussis vaccine 8–12 weeks before delivery provides newborns with the highest antibody titer [15], similar to what we have observed for the highest levels of passively acquired antibodies from COVID-19 vaccination during the third trimester.

Our study adds to current understandings by demonstrating that second- and third-trimester COVID-19 vaccines were associated with a higher neutralization against the Wuhan and Omicron variants, as shown by the multivariable analysis (Table 2). Considering women who are at high risk for preterm labor, such as those with multifetal pregnancies, the preterm pre-labor rupture of membranes, or placenta previa, an earlier booster dose during pregnancy, specifically in the second trimester, may be worth considering. This aligns with recent recommendations from the World Health Organization (WHO), the American College of Obstetricians and Gynecologists (ACOG), the Society for Maternal Fetal Medicine (SMFM), and the Center for Disease Control and Prevention (CDC) guidelines [19,21,31].

Increases in cord sera neutralization for both the Wuhan and Omicron strains were observed with second- and third-trimester vaccines, but not with maternal Omicron infection in the multivariate analysis. Maternal Omicron infection, on the other hand, increased the levels of cord anti-S antibodies in the multivariate analysis. We believe the difference in neutralization results from the antibody titers, reflecting the possible limitations in the direct measurement of antibody titers only [32], and the relative high neutralization levels for current vaccines reported in previous studies [33].

In the multivariable analysis (Table 2), maternal Omicron infection significantly increased the anti-S levels in both maternal and umbilical cord blood. However, the impact on Omicron neutralization was observed only in maternal blood, not in cord blood. This discrepancy may be time-related, as the past literature indicates that the expression of neutralizing antibodies relative to anti-S antibodies requires temporal maturation after infection [29]. Moreover, we concurrently observed a relative time lag of the rise in anti-S and neutralization potencies in umbilical cord blood when compared to its maternal counterpart (Figure 4 and Figure 5).

Peaks of maternal anti-S antibodies were observed at around 8 weeks after antigen exposure, similar to previous studies [34,35]. In our study, a delay in the elevation of cord serum anti-S antibody levels was observed in comparison to maternal serum levels, peaking at 8–12 weeks after vaccination or infection (Figure 4). Consequently, it can be inferred that administering the vaccine during the second or third trimester of pregnancy, specifically 8–12 weeks before full term, may yield the most ideal results.

First-generation vaccines resulted in higher neutralization potency for the Wuhan strain compared to the Omicron strain (Figure 5). Importantly, these vaccines continue to offer significant benefits for neonatal passive immune protection against the novel Omicron variant. Similar to the temporal dynamics observed for anti-S antibodies (Figure 4), a delayed decline in cord sera, when compared to their maternal counterparts, was evident for both Wuhan and Omicron neutralization (Figure 5).

Our study revealed that the transplacental ratios of both anti-S and anti-N antibodies in maternal–fetal pairs had a positive correlation with the time interval from the last antigen exposure, either vaccination or infection, within 100 days (Figure 6). We also confirmed a negative correlation between antenatal COVID-19 infection and transplacental anti-S antibody transfer ratio by multivariable regression (Table A1). We validated the assumption of COVID-19 infection’s negative effect on transplacental antibody transfer. Similarly, concurrent infections during pregnancy, such as malaria, dengue virus, or human immunodeficiency virus infection, can additionally affect the placental integrity and antibody transfer [36,37].

Our study presents some significant advantages. First, our carefully selected study population was unique in that it enabled us to analyze only patients infected with the Omicron variant and without any previous COVID-19 exposure. All participants in the control group were confirmed by negative anti-N results. Second, we were able to confirm the beneficial effect of vaccines during the second and third trimesters towards both the original vaccine-targeted Wuhan strain and the novel Omicron variant by including participants with vaccine injections across all three trimesters. Third, an optimal interval from antigen exposure to delivery (8–12 weeks) was identified to maximize neonatal protection. Fourth, the negative effect of COVID-19 infection on transplacental SARS-CoV-2 antibody transfer was confirmed [13,38].

However, our study has some limitations. The sample size was relatively small, and our study cohort was meticulously chosen to mitigate potential confounders, restricting the generalizability of our findings. The exclusion of patients with preterm labor and the inability to assess immunogenicity in pregnant individuals with autoimmune diseases contribute to these constraints. Two non-infected participants with autoimmune diseases were identified, one with systemic lupus erythematosus and one with antiphospholipid syndrome, and showed varying immunogenicity. The participant with systemic lupus erythematosus received her last vaccine in the third trimester and had unremarkable immunogenicity comparing to the rest of the cohort. The one with antiphospholipid syndrome had her last vaccine in the second trimester and had below-average immunogenicity when compared to her group. Yet, the small sample size precludes definitive conclusions. Autoimmune diseases are recognized as potential immunological factors [39,40], adding complexity to our understanding of this study’s outcomes.

Furthermore, a relatively higher Cesarean section rate was observed in the control group, as participants from the control group were frequently admitted to the tertiary center due to high-risk pregnancies. Recognizing this as a potential confounder, we aimed to mitigate its impact by selecting participants who delivered at term. While the baseline characteristics between the case and control groups were similar, differences among the two groups may still pose as potential confounders in our study.

Additionally, the timing of SARS-CoV-2 infection in our study was predominantly in the third trimester, often close to the time of delivery, with a median interval of 4.5 days. Consequently, we were unable to assess the durability of antibodies or the transplacental antibody transfer ratio following infection over time. In the infection group, all vaccinations were administered before the infection, with a mean interval of 129 days between the last vaccination and Omicron infection. To analyze the impacts of infection and vaccination separately, we conducted multivariable regression for antibody levels and neutralization towards the Wuhan and Omicron strains. Surprisingly, we found that maternal Omicron infection, while enhancing protection against the Omicron strain in maternal blood, did not provide assistance to umbilical cord blood against the Omicron strain in our study. Conversely, the first-generation vaccine, regardless of the trimester in which it was administered during pregnancy, exhibited a positive protective effect on umbilical cord blood. This again reinforces the importance of administering COVID-19 vaccines during pregnancy.

Maternal-acquired passive immunity includes not only immunoglobulin but also cellular responses, such as the vertical transfer of pathogen-specific T cells. Since our focus was solely on the measurable immunoglobulin titers and its neutralizing potencies in maternal and cord serum, the complex immune response following infection or vaccines at the cellular level may also be dismissed [32]. Previous studies have demonstrated many factors which possibly influence the transplacental antibody transfer efficacy, for instance, a better placental function may contribute to increased transplacental antibody transfer [41]. The molecular explanation for the decreased transplacental transfer of IgG in antenatal COVID-19, including whether the trophoblast Fc glycosylation and FCGR3A expression have a role, remains to be explored [42]. Further research can help to clarify other factors which influence transplacental antibody transfer, such as the gestational age at birth, individual placental function, and the specific affinity of placental receptors for antibodies against different pathogens [43].

We found that a booster dose of the COVID-19 vaccine during both the second and third trimesters can provide transplacental humoral protection against both the Wuhan wild-type strain and Omicron variant, and that COVID-19 infection had a negative influence on the transplacental transfer ratio of SARS-CoV-2 antibodies.

## 5. Conclusions

Our findings showed that the level of cord antibodies peaked at around 8–12 weeks after either vaccination or infection. Third-trimester vaccines boosted anti-S level significantly. However, both second and third trimester vaccination provides transplacental protection against both the vaccine-covered strain and a novel, vaccine-uncovered variant, as confirmed by neutralization tests. A booster dose during either the second or third trimester is encouraged for maximum transplacental protection.

## Figures and Tables

**Table 1:** Characteristics of pregnant women with and without COVID-19.

Characteristics	COVID-19 Positive (*n* = 38)	COVID-19 Negative (*n* = 37)	*p* Value
Age, years	33 (5.4) ^1^	33 (5.7) ^1^	0.984 ^1^
Body mass index, kg/$m^{2}$	26.5 (3.6) ^1^	27.1 (4.4) ^1^	0.497 ^1^
Gravida			0.274
Nulliparous	21 (55)	25 (68)	
Multiparous	17 (45)	12 (32)	
Gestational Age at Birth, weeks	38.9 (0.8) ^2^	38.4 (1.3) ^2^	0.245 ^2^
Mode of delivery			0.012
Vaginal delivery	30 (79)	19 (51)	
Cesarean section	8 (21)	18 (49)	
Obstetric complications			
Hypertensive disorder	5 (13)	7 (19)	0.496
Gestational diabetes mellitus	3 (8)	4 (11)	0.711 ^3^
COVID-19 Omicron infection to delivery, days	4.5 (6) ^1^		
Timing of COVID-19 Omicron infection			
First trimester, <13 weeks	0 (0)		
Second trimester, 13–27 weeks	3 (8)		
Third trimester, ≥27 weeks	35 (92)		
Vaccine dose during pregnancy			
First trimester, <13 weeks	18 (47)	7 (19)	0.029
Second trimester, 13–27 weeks	19 (50)	17 (46)	0.902
Third trimester, ≥27weeks	13 (8)	11 (30)	0.500
Vaccine dose			
Primary series with one booster dose	22 (58)	22 (60)	0.891
Primary series, completed	13 (34)	12 (32)	0.870
Primary series, partial	3 (8)	3 (8)	1.000 ^3^
Vaccine Type			0.924 ^3^
Messenger RNA	12 (32)	10 (27)	
Adenoviral vector	2 (5)	3 (8)	
Protein subunit	2 (5)	1 (3)	
Mixed vaccine	22 (58)	23 (62)	
Vaccine Administered During Pregnancy, doses			
Messenger RNA	42 (84)	30 (86)	0.533
Adenoviral vector	6 (12)	5 (14)	0.781
Protein subunit	2 (4)	0 (0)	0.493 ^3^
Last vaccine to maternal blood sampling, days	143.8 (90.7) ^1^	142.2 (97.5) ^1^	0.941 ^1^
Last vaccine to delivery, days	145.7 (91.6) ^1^	143 (97.8) ^1^	0.902 ^1^
Last vaccine to COVID-19 Omicron infection, days	129 (90.4) ^1^		

Data shown as numbers (percentages). Statistical significance was determined by the chi-square test. ^1^ Data shown as means (standard deviation). Statistical significance was determined by the *t* test. ^2^ Data shown as medians (quartile deviation). Statistical significance was determined by the Mann–Whitney U test. ^3^ Calculated by fisher’s exact test due to >20% of cells having expected cell counts less than 5. Abbreviations: COVID-19, coronavirus disease (COVID-19).

**Table 2:** Multivariable linear regression analysis of factors influencing the concentration of anti-S antibody and response rate of neutralizing antibodies.

	Maternal Blood, Anti-S Antibody (U/mL)			Cord Blood, Anti-S Antibody (U/mL)		
Predictor Variables	B	SE	*p* Value	B	SE	*p* Value
COVID-19 Omicron infection	11,096.887	3895.534	0.006	7797.896	3433.965	0.026
First trimester vaccines	7,587.933	4409.352	0.090	6053.706	3886.902	0.124
Second trimester vaccines	−2730.440	3998.881	0.497	102.250	3525.067	0.977
Third trimester vaccines	4281.695	4383.091	0.332	9838.372	3863.753	0.013
	Maternal blood, Wuhan strain ^1^			Cord blood, Wuhan strain ^1^		
Predictor variables	B	SE	*p* value	B	SE	*p* value
COVID-19 Omicron infection	3.421	5.339	0.524	0.022	6.451	0.997
First trimester vaccines	8.648	6.043	0.157	7.091	7.302	0.222
Second trimester vaccines	25.789	5.481	<0.001	27.066	6.622	<0.001
Third trimester vaccines	27.152	6.007	<0.001	22.226	7.258	<0.001
	Maternal blood, Omicron strain ^1^			Cord blood, Omicron strain ^1^		
Predictor variables	B	SE	*p* value	B	SE	*p* value
COVID-19 Omicron infection	16.150	6.451	0.015	2.507	5.080	0.700
First trimester vaccines	12.414	7.302	0.094	17.383	5.750	0.021
Second trimester vaccines	18.330	6.622	0.007	29.676	5.214	<0.001
Third trimester vaccines	33.051	7.258	<0.001	37.060	5.715	<0.001

Abbreviations: B, estimated effect on outcome variable per predictor variable; SE, standard error; COVID-19, coronavirus disease (COVID-19). ^1^ Response rate of neutralizing antibodies in maternal or cord blood, %.

## Data Availability

The raw data supporting the conclusions of this article will be made available by the authors upon request.

## Supplementary Materials

The following supporting information can be downloaded at: https://www.mdpi.com/article/10.3390/vaccines12020164/s1, Table S1: Comparison of maternal anti-S antibody concentration, cord anti-S antibody concentration, and response rate of neutralizing antibodies against Wuhan strain and Omicron variant, between participants receiving latest vaccine in different trimester; Table S2: *p*-values for post-hoc multiple comparisons of maternal anti-S antibody concentration, cord anti-S antibody concentration, between participants receiving latest vaccine in different trimester; Table S3: Anti-spike protein receptor-binding domain (*S*-*RBD*) concentrations and antibody transfer ratio in maternal and cord blood among maternal-infant dyads without COVID-19, with COVID-19 noted for ≤7 days, and with COVID-19 noted for >7 days.

## Appendix A

**Table A1:** Multivariable linear regression analysis of factors influencing the concentration of Transplacental ratios of anti-spike protein (S) antibody.

	Transplacental Transfer Ratio of Anti-S Antibody		
Predictor Variables	B	SE	*p* Value
COVID-19 infection	−0.605	0.166	<0.001
Vaccine during first trimester	−0.053	0.188	0.779
Vaccine during second trimester	0.583	0.171	0.001
Vaccine during third trimester	0.127	0.187	0.501

Abbreviation: B, estimated effect on outcome variable per predictor variable; SE, standard error; COVID-19, coronavirus disease 2019.
