# Supplementary material for: Impact of Antenatal SARS-CoV-2 Exposure on SARS-CoV-2 Neutralization Potency
Source: Vaccines (Basel). 2024 Feb 5;12(2):164. doi: 10.3390/vaccines12020164 (PMC10892697; doi:10.3390/vaccines12020164)
Supplement: Supplementary file 1 [file vaccines-12-00164-s001.zip › vaccines-2812762-supplementary.pdf]

Supplementary Materials

Table S1. Comparison of maternal anti-S antibody concentration, cord anti-S antibody concentration, and response rate of neutralizing antibodies against Wuhan strain and Omicron variant, between participants receiving latest vaccine in different trimester.

All participants (*n*=75)				
	T1[4] (*n*=25)	T2[5] (*n*=26)	T3[6] (*n*=24)	*P* value
Anti-S, maternal blood (U/mL)[1]	578.50 (160.25-6425.00)	4565.00 (1749.00-10,213.25)	10,627.50 (6102.75-18,021.50)	<0.001
Anti-S, cord blood (U/mL)	576.10 (184.00-3757.00)	6017.00 (3838.75-11,717.25)	16,082.50 (10,805.50-19,823.75)	<0.001
Maternal Wuhan (%)[2]	52.00 (38.46-97.51)	97.78 (96.13-98.15)	98.15 (98.05-98.22)	<0.001
Maternal Omicron (%)[3]	17.15 (8.36-73.17)	67.195 (44.46-85.09)	86.71 (65.02-93.69)	<0.001
Cord Wuhan (%)	59.48 (47.25-97.74)	97.875 (97.42-98.24)	98.15 (98.01-98.22)	<0.001
Cord Omicron (%)	22.50 (10.90-66.91)	79.18 (57.37-87.63)	89.12 (78.27-94.45)	<0.001
Control group: Participants without COVID-19 infection (*n*=37)				
	T1[4] (*n*=12)	T2[5] (*n*=14)	T3[6] (*n*=11)	*P* value
Anti-S[1], maternal blood (U/mL)	264.15 (56.60-1075.98)	2035.50 (1070.93-4912.75)	10,442.00 (9535.00-13,033.00)	<0.001
Anti-S, cord blood (U/mL)	269.80 (74.54-1819.75)	5937.50 (2932.33-11,280.75)	14,999.00 (12,085.00-19,042.00)	<0.001
Maternal Wuhan (%)[2]	48.99 (24.82-91.00)	96.88 (84.49-97.91)	98.15 (98.04-98.19)	<0.001
Cord Wuhan (%)	51.13 (29.51-97.45)	97.65 (97.10-98.25)	98.15 (98.04-98.22)	<0.001
Maternal Omicron (%)[3]	20.73 (15.64)[7]	50.53 (23.81)[7]	79.27 (14.71)[7]	<0.001[7]
Cord Omicron (%)	19.88 (13.61-30.66)	76.45 (46.09-85.85)	88.94 (79.36-91.98)	<0.001
Case group: Participants with COVID-19 infection (*n*=38)				
	T1[4] (*n*=13)	T2[5] (*n*=12)	T3[6] (*n*=13)	*P* value
Anti-S, maternal blood (U/mL)	6395.00 (348.35-27,936.50)	6076.50 (2630.50-25,784.25)	15,621.00 (5102.00-34,328.00)	0.564
Anti-S, cord blood (U/mL)	1328.00 (332.55-30,385.00)	6219.50 (4186.25-26,469.00)	18,958.00 (8727.50-30,972.00)	0.231
Maternal Wuhan (%)	97.32 (39.16-98.21)	98.13 (97.30-98.21)	98.15 (97.90-98.30)	0.146
Cord Wuhan (%)	88.52 (56.86-98.13)	98.10 (97.50-98.23)	98.15 (97.72-98.21)	0.090
Maternal Omicron (%)[3]	54.65 (42.02)[7]	78.78 (19.94)[7]	79.43 (19.61)[7]	0.064[7]
Cord Omicron (%)	34.18 (7.99-96.91)	79.80 (61.23-96.44)	91.27 (71.35-96.64)	0.193

Notes: Data are shown as medians (interquartile ranges). Statistical significance was determined by the Kruskal-Wallis test.

[1]Concentration of anti-spike protein antibody concentration, U/mL.

[2]Response rate of neutralizing antibodies against Wuhan strain, %.

[3]Response rate of neutralizing antibodies against Omicron variant, %.

[4]T1, last vaccine before pregnancy and during first trimester
[5]T2, last vaccine in second trimester
[6]T3, last vaccine in third trimester
[7]Data are shown as means (standard deviation). Statistical significance was determined by ANOVA.
Abbreviation: COVID-19, coronavirus disease (COVID-19); Anti-S, anti-spike protein antibody.

Table S2. P-values for post-hoc multiple comparisons of maternal anti-S antibody concentration, cord anti-S antibody concentration, between participants receiving latest vaccine in different trimester

All participants (*n*=75)

	T1[4]-T2[5]	T2-T3[6]	T1-T3
Maternal Anti-S[1]	0.019	0.013	<0.001
Cord Anti-S	0.002	0.006	<0.001
Maternal Wuhan[2]	0.006	0.01	<0.001
Cord Wuhan	<0.001	0.173	<0.001
Maternal Omicron[3]	0.005[ǂ]	0.14[ǂ]	<0.001[ǂ]
Cord Omicron	<0.001	0.048	<0.001

Control group: Participants without COVID-19 infection (*n*=37)

	T1-T2	T2-T3	T1-T3
Maternal Anti-S	0.002	<0.001	<0.001
Cord Anti-S	<0.001	0.006	<0.001
Maternal Wuhan	0.005	<0.001	<0.001
Cord Wuhan	0.003	0.095	<0.001
Maternal Omicron	0.143[ǂ]	1.000[ǂ]	0.115[ǂ]
Cord Omicron	<0.001	0.009	<0.001

Note: Statistical significance was determined by the Mann-Whitney U test after Kruskal-Wallis tests.

[ǂ]Statistical significance was determined by the Bonferroni test after ANOVA tests.

[1]Concentration of Anti-S concentration, U/mL.

[2]Response rate of neutralizing antibodies against Wuhan strain, %.

[3]Response rate of neutralizing antibodies against Omicron variant, %.

[4]T1, last vaccine before pregnancy and during first trimester

[5]T2, last vaccine in second trimester

[6]T3, last vaccine in third trimester

Abbreviation: COVID-19, coronavirus disease (COVID-19); Anti-S, anti-spike protein antibody.

Table S3. Anti-spike protein receptor-binding domain concentrations in maternal and cord blood among maternal-infant dyads without COVID-19, with COVID-19 noted for ≤ 7 days, and with COVID-19 noted for > 7 days.

Variables	COVID-19			*P* value
	COVID- (*n*=37)	COVID+, ≤ 7 days (*n*=26)	COVID+, > 7 days (*n*=12)	
Anti-S, maternal blood (U/mL)	2070.00 (314.90-9706.00)	5833.00 (850.30-11,016.80)	29,256.50 (14,403.80-47,773.50)	<0.001*
Anti-S, cord blood (U/mL)	5317.00 (545.60-12,393.50)	6420.00 (612.10-16,190.50)	33,017.50 (5599.50-49,075.50)	0.008[ǂ]

Note:

*Data are shown as means (standard deviation). Statistical significance was determined by One-way ANOVA.

[ǂ]Data are shown as medians (interquartile ranges). Statistical significance was determined by the Kruskal-Wallis test.

Abbreviation: COVID-19, coronavirus disease (COVID-19); Anti-S, anti-spike protein antibody.
